# Anti-inflammatory effects of *Glycyrrhiza glabra* homeopathic formulations in a rat model of lipopolysaccharide-induced inflammation

**DOI:** 10.1016/j.jaim.2025.101267

**Published:** 2025-11-15

**Authors:** Bharti Gawai, Amol A. Tagalpallewar, Anil T. Pawar, Akshay M. Baheti

**Affiliations:** Department of Pharmaceutical Sciences, School of Health Sciences and Technology, Dr. Vishwanath Karad MIT World Peace University, Pune-411038, Maharashtra, India

**Keywords:** Anti-inflammatory, Cytokines, *Glycyrrhiza glabra*, Homeopathy, Inflammation, Lipopolysaccharide

## Abstract

**Background:**

Inflammation plays a crucial role in the pathogenesis of various chronic diseases. Alternative therapies, such as homeopathy, have gained attention for their potential in managing inflammatory conditions. *Glycyrrhiza glabra*, commonly known as licorice, is well-documented for its anti-inflammatory properties in herbal medicine. However, its efficacy in homeopathic formulations remains largely unexplored.

**Objective:**

This study aimed to evaluate the anti-inflammatory potential of different homeopathic potencies of *Glycyrrhiza glabra* against lipopolysaccharide (LPS)-induced inflammation in rats.

**Methods:**

Male Wistar rats were divided into seven groups: normal control, LPS-induced inflammation control, dexamethasone-treated, *Glycyrrhiza glabra* homeopathic mother tincture (GHMT)-treated, and groups treated with *Glycyrrhiza glabra* homeopathic potency (G6CH, G30CH, G200CH). Anti-inflammatory effects of GHMT, G6CH, G30CH, and G200CH were evaluated against LPS-induced inflammation by measuring paw volume, serum pro-inflammatory cytokine levels [tumor necrosis factor-alpha (TNF-α) and interleukin-6 (IL-6)], levels of oxidative stress biomarkers (superoxide dismutase, glutathione, and catalase) in paw tissues, and histopathological changes in inflamed paw tissues.

**Results:**

The results demonstrated a significant reduction in paw volume in GHMT and G200CH-treated groups (p<0.0001) as compared to the inflammation control group. Additionally, the levels of serum TNF-α and IL-6 were significantly lowered (p<0.0001), and oxidative stress biomarkers showed significant improvement (p<0.0001) in GHMT and G200CH-treated groups. Histopathological examination further confirmed the reversal of inflammation-induced tissue changes by *G. glabra* homeopathic formulations, indicating its anti-inflammatory activity.

**Conclusion:**

These findings provide scientific evidence supporting the anti-inflammatory potential of homeopathic formulations of *Glycyrrhiza glabra*, particularly GHMT and G200CH. The study suggests that these homeopathic potencies could serve as promising alternative anti-inflammatory agents, warranting further research to elucidate the underlying molecular mechanisms.

## 1 Introduction

Inflammation is a key physiological response triggered when the body is injured, infected, or exposed to harmful substances. Pain, heat, swelling, and redness are the classic signs of inflammation, resulting from vascular changes and local immune cell responses [1,2]. The major microcirculatory events during inflammation include increased vascular permeability, migration and accumulation of leukocytes, and the subsequent release of pro-inflammatory mediators [3]. The inflammatory process involves a highly coordinated network of cell types, with activated macrophages, monocytes, and other immune cells playing a pivotal role in responding to tissue injury and infection [4].

Inflammation triggers the release of chemokines and growth factors that attract immune cells, such as neutrophils and monocytes, to the injury site [5]. Injured epithelial and endothelial cells release substances that initiate the inflammatory cascade. To prevent excessive inflammation and restore tissue homeostasis, the body employs counter-regulatory mechanisms, including reduced neutrophil infiltration, cytokine modulation, and macrophage activation to initiate the healing process [6]. In conditions such as rheumatoid arthritis, immune cells (such as T cells and macrophages) accumulate in the affected tissue, leading to chronic inflammation [[7], [8], [9]].

Prolonged exposure to pro-inflammatory cytokines, such as interleukin-1β (IL-1β), tumor necrosis factor-alpha (TNF-α), and interleukin-6 (IL-6) exacerbates inflammation by attracting more immune cells to the affected tissue, worsening conditions such as joint inflammation [10]. Conventional anti-inflammatory therapies often rely on nonsteroidal anti-inflammatory drugs (NSAIDs) or corticosteroids. However, these agents may lead to adverse effects with long-term use [11]. As a result, there is growing interest in alternative approaches that modulate the inflammatory response more safely and holistically. Homeopathy and other alternative medicine systems adopt a holistic approach for patient care, addressing not only the physical symptoms of disease but also the mental, emotional, and spiritual aspects. According to the World Health Organization, homeopathy is one of the most widely practiced medical systems globally [12]. The homeopathic philosophy aims to treat the person by selecting constitution-improving remedies that help to prevent the recurrence of autoimmune diseases without causing further side effects. The guiding principle of homeopathy is *similia similibus curentur*, meaning "like cures like" [13].

Nature has provided a wealth of medicinal substances, particularly plants rich in bioactive phytochemicals. One of these plants is *Glycyrrhiza glabra* Linn. (*G. glabra*), a member of the Leguminosae family, which has been used for its medicinal properties for centuries. *G. glabra* is native to several countries across the globe and is also cultivated in many parts of the world [11]. *G. glabra* holds significant commercial value due to its applications in the food, medicine, cosmetics, and tobacco industries [14].

The root of *G. glabra* contains several bioactive compounds, including glycyrrhizin, glycyrrhetinic acid, isoliquiritigenin, and glycyrrhizic acid. These compounds are known for their anti-inflammatory, anti-asthmatic, anti-cancer, anti-diabetic, anti-microbial, and anti-spasmodic effects. Glycyrrhetinic acid, a key constituent of *G. glabra*, exhibits anti-inflammatory activity similar to that of glucocorticoids. For over 2,000 years, *G. glabra* root extract has been used to treat mouth and stomach ulcers. Recent studies have shown that glycyrrhizic acid inhibits inflammatory markers by suppressing prostaglandin synthesis, cyclooxygenase activity, and platelet aggregation [15,16].

Although the anti-inflammatory potential of *G. glabra* has been well-documented, its effectiveness in homeopathic formulations remains underexplored. As the demand for natural and complementary therapies in the management of inflammation grows, evaluating homeopathic formulations of *G. glabra* is crucial. Lipopolysaccharide (LPS), a major component of the outer membrane of gram-negative bacteria, is widely used to induce experimental models of inflammation due to its potent ability to activate immune cells and trigger the release of pro-inflammatory cytokines. The LPS-induced inflammation model in rodents is a well-established for evaluating the efficacy of anti-inflammatory agents, as it closely mimics various aspects of acute and systemic inflammatory responses seen in human disease [17]. Given the importance of identifying safe and effective anti-inflammatory treatments, this study was conducted to evaluate the efficacy of various homeopathic potencies of *G. glabra* against LPS-induced inflammation in rats.

## 2 Materials and methods

### 2.1 Chemicals and reagents

The potencies of 6CH, 30CH, and 200CH (*Dr. Willmar Schwabe* India, Batch No. 0738339) and *G. glabra* mother tincture (*Dr. Willmar Schwabe* India, Batch No. 0478487) were used for the study. Dexamethasone tablets (Tablet Aardex; Cadila Pharma, India; Batch No. IDXG348) and LPS (TCI Chemicals, India, Batch No. L2630) were purchased from an approved vendor. ELISA kits for TNF-α (Feiyue, Batch No. KD060640710002) and IL-6 (Feiyue, Batch No. KD052240710001) were used for the study.

### 2.2 Animals

Wistar rats (200–250 g, aged 8–12 weeks) were used for the study. The animals were procured from Scitesla Private Limited, Navi Mumbai, India. They were housed under standard laboratory conditions and fed a standard laboratory diet provided by VRK Nutritional Solutions, India, with free access to food and water. The study was approved by the Institutional Animal Ethics Committee (Approval No. 2166/PO/RcBi/S/22/CPCSEA).

### 2.3 Administration of doses

Using sterile water, 0.1 ml of *G. glabra* homeopathic mother tincture and potencies of 6CH, 30CH, and 200CH (GHMT, G6CH, G30CH, and G200CH respectively) were diluted to 1 ml and administered orally to respective treatment groups. LPS was injected 1 h before and 4 h after the administration of GHMT and its different potencies. One hour before the LPS-induced inflammation, the standard control anti-inflammatory medication dexamethasone (0.5 mg/kg, p.o.) was administered.

### 2.4 Experimental design

Anti-inflammatory activity of homeopathic potencies of *G. glabra* was evaluated using LPS-induced inflammation in rats [18]. Rats were randomly divided into seven groups, with six in each group. Group 1 was the normal control (NC) group and did not receive any treatment. Inflammation was induced in Groups 2 to 7 by subcutaneous injections of 100 μg LPS solution into the subplantar surface of the right hind paw [19]. Group 2 was the disease control (DC) group and did not receive any treatment. Group 3 was the standard control (SC) group and was treated with 0.5 mg/kg of dexamethasone [20]. Groups 4 to 7 were *G. glabra* homeopathic formulations-treated groups and received 0.1 ml of G6CH, G30CH, G200CH, and GHMT, respectively. Dexamethasone and *G. glabra* homeopathic formulations were administered to overnight-fasted rats twice daily for 28 days via oral route.

### 2.5 Measurement of paw volume

The paw volume was measured using a plethysmometer (Orchid Scientific Instruments, India; Model No. PLM 01 PLUS) at days 0, 7, 14, 21 and 28 of the treatment period.

### 2.6 Serum estimations

After the paw volume measurement on the 28th day, blood was collected via the retro-orbital route under ether anesthesia. The blood samples were centrifuged at 10,000 rpm for 15 min to obtain the serum. The serum levels of TNF-α and IL-6 were measured using ELISA kits.

### 2.7 Tissue homogenate analysis

After blood collection, rats were sacrificed by an overdose of anesthesia, and inflamed paw tissues were isolated for biochemical estimation. Isolated tissues were homogenized in 0.1 M phosphate buffer (pH 7.4). The tissue homogenate was centrifuged at 10,000 rpm for 30 min, and the supernatant was analyzed for the levels of pro-inflammatory cytokines, lipid peroxidation (MDA), and antioxidants (SOD, CAT, and GSH) [[21], [22], [23], [24]].

### 2.8 Histopathology

The isolated inflamed paw tissues were also subjected to histopathological analysis. Paw tissues were fixed in 10% neutral buffered formalin, processed through a series of graded alcohol and xylene, embedded in paraffin wax, sectioned at 5 μm, stained with hematoxylin and eosin (H&E), and examined under a light microscope to detect any alterations in tissue histoarchitecture.

### 2.9 Statistical analysis

The statistical analysis was performed using GraphPad Prism 8.0.2 software. Mean±Standard error of mean (SEM) was used to express the values. The normality of the data was checked using the Shapiro-Wilk test, which confirmed that all datasets followed a normal distribution (p>0.05). Statistical significance between the disease control group and the normal control group was calculated using Student's t-test, whereas one-way analysis of variance (ANOVA) followed by Dunnett's comparison test was used to compare the drug-treated groups to the disease-control group. Significant differences were defined as p<0.05.

## 3 Results

### 3.1 Effect on paw volume

A significant increase in paw volume (p<0.0001) was observed in the DC group by Day 7, indicating the onset of LPS-induced inflammation. In contrast, the groups treated with the *G. glabra* formulations showed a reduction in paw volume beginning on Day 7, with marked improvements by Day 28. In particular, the GHMT, G30CH, and G200CH groups exhibited significant anti-inflammatory effects, with paw volumes comparable to the standard control dexamethasone. By Day 28, paw inflammation had nearly subsided in the treated groups, with results showing that GHMT (p<0.0001), G6CH (p<0.0001), G30CH (p<0.0001), and G200CH (p<0.0001) were particularly effective (Table 1).

**Table 1:** Effects of *G. glabra* homeopathic formulations on paw volume.

Sr. No.	Groups	Paw volume (ml)				
		Day 0	Day 7	Day 14	Day 21	Day 28
1	Normal Control	1.74±0.02	1.77±0.02	1.80±0.02	1.83±0.02	1.85±0.02
2	Disease Control	1.76±0.04	2.90±0.02∗∗∗∗	3.78±0.03∗∗∗∗	3.52±0.04∗∗∗∗	3.20±0.03∗∗∗∗
3	Standard Control	1.76±0.04	2.52±0.03∗∗∗∗	2.26±0.02∗∗∗∗	2.07±0.02∗∗∗∗	2.01±0.02∗∗∗∗
4	GHMT	1.76±0.02	2.42±0.02∗∗∗∗	2.31±0.02∗∗∗∗	2.11±0.01∗∗∗∗	1.97±0.02∗∗∗∗
5	G6CH	1.84±0.02	2.87±0.02	2.90±0.02∗∗∗∗	2.72±0.02 ∗∗∗∗	2.65±0.02∗∗∗∗
6	G30CH	1.85±0.02	2.89±0.03	2.70±0.02∗∗∗∗	2.51±0.03∗∗∗∗	2.30±0.03∗∗∗∗
7	G200CH	1.86±0.02	2.73±0.02∗∗∗	2.52±0.02∗∗∗∗	2.33±0.01∗∗∗∗	1.98±0.01∗∗∗∗

Values are Mean±SEM; N=6; ∗p<0.05, ∗∗p<0.01, ∗∗∗p<0.001, ∗∗∗∗p<0.0001. Disease control vs. normal control and drug-treated groups vs. disease control.

### 3.2 Effect of serum cytokine levels

Elevated levels of TNF-α and IL-6 cytokines were found in the DC group (p < 0.0001) as compared to the NC control. Interestingly, treatment with *G. glabra* formulations led to a significant reduction in serum TNF-α and IL-6 levels. The GHMT, G6CH, G30CH, and G200CH groups showed a significant decrease in cytokine levels, with results comparable to the group treated with dexamethasone (Table 2).

**Table 2:** Effects of *G. glabra* homeopathic formulations on serum TNF-α and IL-6 levels.

Sr. No.	Groups	TNF-α (pg/ml)	IL-6 (pg/ml)
1	Normal Control	20.70±0.51	246.28±5.22
2	Disease Control	320.15±18.73∗∗∗∗	980.23±16.71∗∗∗∗
3	Standard Control	78.30±2.30∗∗∗∗	399.76±10.99∗∗∗∗
4	GHMT	68.62±1.00∗∗∗∗	417.93±23.36∗∗∗∗
5	G6CH	142.43±2.16∗∗∗∗	674.42±4.64∗∗∗∗
6	G30CH	122.31±1.57∗∗∗∗	558.56±6.09∗∗∗∗
7	G200CH	101.52±3.51∗∗∗∗	458.02±2.55∗∗∗∗

Values are Mean±SEM; N=6; ∗p<0.05, ∗∗p<0.01, ∗∗∗p<0.001, ∗∗∗∗p<0.0001. Disease control vs. normal control and drug-treated groups vs. disease control.

### 3.3 Effect on oxidative stress parameters

The disease control group showed significant decrease in antioxidant enzymes (SOD, GSH, and CAT) along with an increase in levels of lipid peroxidation (MDA). These changes were significantly improved by treatment with the GHMT, G30CH, and G200CH (Table 3).

**Table 3:** Effects of *G. glabra* homeopathic formulations on oxidative stress parameters in tissue homogenate.

Sr. No.	Groups	GSH (mM/g Tissue)	SOD (U/mg protein)	Catalase (U/mg protein)	Lipid peroxidation-MDA (nmol/mg protein)
1	Normal Control	0.25±0.015	25.13±0.38	4.13±0.05	2.78±0.04
2	Disease Control	0.14±0.002∗∗∗∗	12.76±0.33∗∗∗∗	1.07±0.03∗∗∗∗	12.55±0.11∗∗∗∗
3	Standard Control	0.24±0.008∗∗∗∗	21.64±0.48∗∗∗∗	3.60±0.05∗∗∗∗	3.53±0.02∗∗∗∗
4	GHMT	0.19±0.010∗∗∗∗	22.76±0.17∗∗∗∗	3.47±0.05∗∗∗∗	3.36±0.03∗∗∗∗
5	G6CH	0.16±0.003	15.98±0.30∗∗∗∗	2.42±0.05∗∗∗∗	5.96±0.04∗∗∗∗
6	G30CH	0.18±0.001∗∗∗	17.82±0.20∗∗∗∗	2.87±0.02∗∗∗∗	4.56±0.02∗∗∗∗
7	G200CH	0.21±0.008∗∗∗∗	22.77±0.43∗∗∗∗	3.19±0.05∗∗∗∗	3.82±0.04∗∗∗∗

Values are Mean±SEM; N=6; ∗p<0.05, ∗∗p<0.01, ∗∗∗p<0.001, ∗∗∗∗p<0.0001. Disease control vs. normal control and drug-treated groups vs. disease control.

### 3.4 Effect on histopathology of paw tissue

**Fig. 1:**
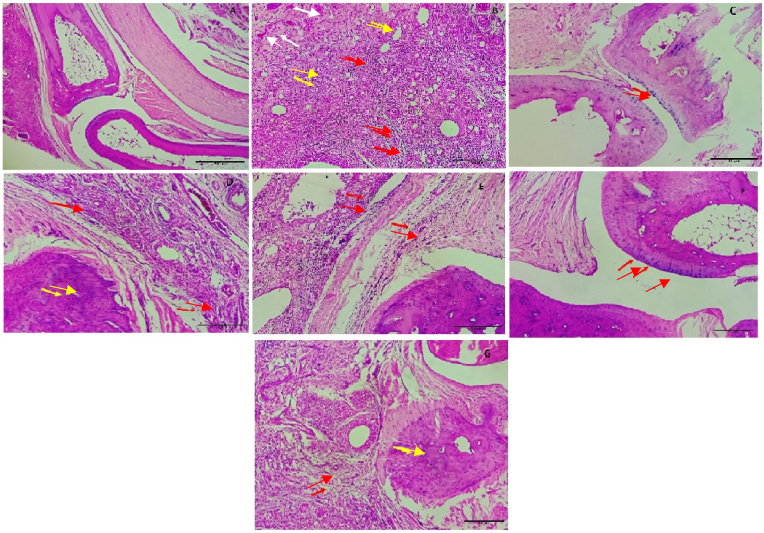
Histopathological images of paw tissues from rats of (A) normal control showing normal paw tissue architecture, (B) disease control indicating hyperaemia and congestion in addition to elevated inflammation edema, infiltration of inflammatory cells, lack of synovial tissue, and alterations in bone and cartilage, (C) dexamethasone (0.5 mg/kg, p.o.)-treated group, and (D, E, F, and G) *G. glabra* homeopathic formulations GHMT G6CH, G30CH, and G200CH-treated groups respectively, showing mild pathological changes (H&E400X). Histopathological sections showing hyperaemia/congestion (yellow arrow), edema (white arrow), and inflammatory cell infiltration (red arrow).

In the disease control group, paw tissue sections showed significant inflammation, characterized by edema, hyperemia, and infiltration of inflammatory cells. There were also notable changes in synovial tissue, bone, and cartilage, indicative of severe inflammatory damage. In contrast, the GHMT and G200CH treatment groups exhibited minimal to mild changes in tissue structure, with reduced edema, inflammatory cell infiltration, and hyperemia. These groups showed near-normal tissue histoarchitecture, with preserved synovial tissue and minimal damage to bone and cartilage. (Fig. 1, Table 4).

**Table 4:** Observations of histopathological images of paw tissues.

Rat code	Hyperaemia/congestion (yellow arrow)	Edema (White arrow)	Inflammatory cell infiltration (Red arrow)	Synovial changes	Cartilage changes	Bone changes	Grade of inflammation^a^
A (Normal Control)	Nil	Nil	Nil	Not seen	Not seen	Not seen	00
B (Disease Control)	+	+++	+++	Not seen	Not seen	Not seen	++++
C (Standard Control)	+	Nil	+	Not seen	Not seen	Not seen	0 to +
D (GHMT)	+	+	+++	Not seen	Not seen	Not seen	+ to ++
E (G6CH)	++	Nil	++	Not seen	Not seen	Not seen	++ to +++
F (G30CH)	+	Nil	+++	Not seen	Not seen	Not seen	++ to +++
G (G200CH)	Nil	Nil	++	Not seen	Not seen	Not seen	++

a Grading: Minimal (+), mild (++), moderate (+++), Severe (++++).

## 4 Discussion

Inflammation is a complex biological response to harmful stimuli, such as pathogens and irritants. It is characterized by increased vascular permeability, immune cell recruitment, and the release of inflammatory mediators. In the current study, we induced inflammation in rats using lipopolysaccharide (LPS), which is a bacterial endotoxin known to trigger a strong immune response, and evaluated the anti-inflammatory effects of various potencies of *G. glabra* homeopathic formulations (GHMT, G6CH, G30CH, and G200CH). The anti-inflammatory activity was assessed by measuring changes in paw volume, which reflects the degree of inflammation.

LPS induces inflammation by binding to Toll-like receptor 4 (TLR4), leading to the activation of nuclear factor kappa B (NF-κB) and subsequent production of pro-inflammatory cytokines such as TNF-α and IL-6. These cytokines, in turn, contribute to swelling, heat, redness, and pain, which are hallmark symptoms of inflammation [25]. There was a significant reduction in the paw volume of LPS-induced inflammation after treatment with *G. glabra* formulations. This suggests that the homeopathic formulations are effective in modulating the inflammatory response and reducing edema. This observation is consistent with the known anti-inflammatory properties of *G. glabra*, which contains bioactive compounds such as glycyrrhizin, glabridin, and isoliquiritigenin. These compounds have been shown to exert anti-inflammatory effects by inhibiting key enzymes like cyclooxygenase (COX), reducing prostaglandin synthesis, and modulating the release of inflammatory cytokines [26]. The potent anti-inflammatory activity observed at the higher concentration of *G. glabra* formulations i.e. homeopathic mother tincture (GHMT), is particularly notable, which indicates that higher doses offer substantial therapeutic benefits, similar to or even surpassing the effects of dexamethasone.

Cytokines such as TNF-α and IL-6 play pivotal roles in the inflammatory cascade, acting as key signaling molecules that amplify the immune response during inflammation. The LPS-induced rise in the serum TNF-α and IL-6 levels was reduced by the *G. glabra* formulations. The reduction in cytokine levels is clinically important, as TNF-α and IL-6 are major drivers of inflammation and tissue damage in various inflammatory diseases. Previous studies have shown that *G. glabra* and its components, particularly glycyrrhizin, can inhibit the production of TNF-α and IL-6 by suppressing NF-κB activation, a key transcription factor involved in the inflammatory response [25,26]. By targeting these cytokines, *G. glabra* formulations not only mitigate inflammation but also provide protection against further tissue damage caused by excessive immune activation. These findings align with previous studies that demonstrated the ability of *G. glabra* to inhibit various inflammatory mediators [26,27]. These studies reported that *G. glabra* extracts could inhibit COX-2 activity, reduce TNF-α and IL-6 production, and prevent inflammation-induced damage, further supporting the results of our study. In our experiment, the higher potency G200CH and mother tincture GHMT formulations effectively reduced inflammation and edema, likely due to their enhanced ability to modulate the immune response and inhibit inflammatory pathways.

Oxidative stress is closely linked to inflammation as the overproduction of reactive oxygen species during inflammation can lead to tissue damage and exacerbate the inflammatory response. In this study, we measured oxidative stress markers, including SOD, GSH, CAT and MDA, to assess the impact of *G. glabra* formulations on oxidative stress. There was a decrease in antioxidant enzymes (SOD, GSH, and CAT) along with an increase in MDA levels, indicating increased oxidative stress. However, treatment with GHMT, G30CH, and G200CH formulations significantly improved these parameters, restoring them to normal levels. *G. glabra* formulations GHMT and G200CH, in particular, were highly effective in reducing oxidative stress, suggesting that these formulations not only counteract inflammation but also provide antioxidant protection. The ability of *G. glabra* to enhance antioxidant defences and reduce oxidative stress is well-established. Previous studies reported that glycyrrhizin, the primary active component of *G. glabra*, can scavenge free radicals and upregulate the expression of antioxidant enzymes, thereby reducing oxidative damage [28]. Our results are consistent with these findings, showing that the homeopathic formulations of *G. glabra* effectively mitigate oxidative stress in LPS-induced inflammation.

There was significant improvement in inflammation-induced histopathological changes in the paw tissue after treatment with *G. glabra* formulations. The GHMT and G200CH groups showed the highest improvement in inflammation-induced pathological changes of paw tissue which highlights the potential of these formulations to promote tissue repair and resolution of inflammation. By reducing both inflammatory markers and oxidative stress, *G. glabra* not only mitigates the acute inflammatory response but also facilitates long-term healing and restoration of tissue integrity**.**

## 5 Conclusion

The study demonstrates the significant anti-inflammatory potential of *G. glabra* homeopathic formulations (mother tincture and its potency) against LPS-induced inflammation in rats. The results revealed notable reductions in inflammation-induced paw volume, serum pro-inflammatory cytokines (TNF-α and IL-6) levels, and oxidative stress marker (MDA) level in paw tissue, as well as improved levels of paw tissue antioxidant enzymes (SOD, GSH, and CAT) and histopathological outcomes by treatment with *G. glabra* homeopathic formulations. This supports the traditional use of *G. glabra* in homeopathy as an effective anti-inflammatory treatment, offering a promising alternative to conventional anti-inflammatory therapies.

## Authors contributions

AMB: Conceptualization, formal analysis, supervison, validation writing – review & editing ATP: Conceptualization, data curation, formal analysis, methodology, software, validation, supervision, writing – review & editing AAT: Conceptualization, visualization, writing – review & editing BG: Writing – original draft, investigation, data acquisition, drafting manuscript, statistical analysis, funding, final approval.

## Declaration of generative AI in scientific writing

The authors declare that they have not used artificial intelligence (AI)-tools for writing and editing the manuscript.

## Funding sources

None

## Conflict of interest

None
